# Frankfurt concept of early inpatient rehabilitation after cochlear implant treatment: Feasibility for aftercare

**DOI:** 10.1007/s00106-024-01441-y

**Published:** 2024-04-17

**Authors:** Stefanie Bruschke, Roland Zeh, Uwe Baumann, Silke Helbig, Timo Stöver

**Affiliations:** 1 University Hospital, ENT department, Goethe University Frankfurt, Frankfurt a. M, Germany; 2 MEDIAN Kaiserberg-Klinik, Bad Nauheim, Germany

**Keywords:** Cochlear implantation, Follow-up treatment, Auditory rehabilitation, Rehabilitation outcome, Speech discrimination, Cochleaimplantation, Anschlussheilbehandlung, Hörrehabilitation, Rehabilitationsergebnis, Sprachverstehen

## Abstract

**Background:**

The Association of the Scientific Medical Societies in Germany (AWMF) clinical practice guideline on cochlear implant (CI) treatment, which was updated in 2020, defined the entire process of CI care for the first time. In the present study, the feasibility and results of very early rehabilitation were examined.

**Materials and methods:**

The intervention group (IG) comprised 54 patients in whom rehabilitation was initiated within 14 (maximally 28) days after implantation. Patients with a significantly longer waiting time were included in the control group (CG, *n* = 21). In addition to the start and duration of rehabilitation, the speech intelligibility achieved with CI was recorded at different timepoints within a 12-month period. In addition, questionnaires were used to assess the effort of fitting the CI processor and the patients’ satisfaction with the outcome as well as the timing of the start of rehabilitation.

**Results:**

Median waiting time between implantation and start of rehabilitation was 14 days in the IG and 106 days in the CG; 92.6% of IG patients were able to start rehabilitation within 14 days. The effect of rehabilitation in the IG was 35 and in the CG 25 percentage points (Freiburg monosyllabic test). After 6 and 12 months of CI use, both groups showed comparable results in the test condition in quiet (IG/CG 6 months: 70%/70%; 12 months: 70%/60%, Freiburg monosyllabic test) and in noise (IG/CG 6 months: −1.1 dB SNR/–0.85 dB SNR; 12 months: −0.65 dB SNR/+0.3 dB SNR, Oldenburg sentence test). Hearing quality assessment scores collected by SSQ (Speech, Spatial and Qualities of Hearing Scale) questionnaire showed better scores in the IG at 6 months, which converged to CG scores at 12 months. The IG was significantly more satisfied with the timing of the start of rehab than the CG. All other data obtained from questionnaires showed no differences between the two groups.

**Conclusion:**

A very early start of inpatient rehabilitation after cochlear implantation was successfully implemented. The rehabilitation was completed within 7 weeks of CI surgery. Comparison of speech recognition test results before and after rehabilitation showed a significant improvement. A clear rehabilitation effect can therefore be demonstrated. Inclusion of CI rehabilitation in the German catalog of follow-up treatments is thus scientifically justified and therefore strongly recommended.

## Abstract (de)

**Hintergrund:**

Mit der im Jahr 2020 aktualisierten AWMF-Leitlinie zur Versorgung mit einem Cochleaimplantat (CI) wurde erstmals der gesamte Prozess einer CI-Versorgung definiert. In der vorliegenden Studie wurden die Machbarkeit und die Ergebnisse einer sehr frühen Rehabilitationsmaßnahme (Reha) untersucht.

**Methodik:**

Es wurden 54 Patienten in die Interventionsgruppe (IG) eingeschlossen, bei der die Reha innerhalb von 14 (maximal 28) Tagen nach der Implantation eingeleitet wurde. In eine Kontrollgruppe (KG, *n* = 21) wurden Patienten mit deutlich längerer Wartezeit eingeschlossen. Neben dem Beginn und der Dauer der Reha wurde das mit CI erreichte Sprachverstehen zu verschiedenen Zeitpunkten innerhalb von 12 Monaten erfasst. Zusätzlich wurde mit Fragebögen der Aufwand der Anpassung des CI-Prozessors und die Zufriedenheit der Patienten mit dem Ergebnis sowie dem Zeitpunkt des Beginns der Reha ermittelt.

**Ergebnisse:**

Die Wartezeit zwischen Implantation und Beginn der Reha lag in der IG bei 14 Tagen und in der KG bei 106 Tagen (Mediane). Es konnten 92,6 % der Patienten der IG die Reha innerhalb von 14 Tagen antreten. Der Effekt der Reha lag in der IG bei 35 und in der KG bei 25 Prozentpunkten (Freiburger Einsilbertest). Nach 6 und 12 Monaten (M) CI-Nutzung zeigten beide Gruppen sowohl in der Testbedingung in Ruhe (IG/KG 6M: 70 %/70 %; 12M: 70 %/60 %, Freiburger Einsilbertest) als auch im Störgeräusch (IG/KG 6M: −1,1 dB SNR/–0,85 dB SNR; 12M: −0,65 dB SNR/+0,3 dB SNR, Oldenburger Satztest) vergleichbare Ergebnisse. Die mittels des Fragebogens Speech, Spatial and Qualities of Hearing Scale (SSQ) erfassten Ergebnisse für die Einschätzung der Hörqualität zeigten nach 6 Monaten eine bessere Bewertung in der IG, die sich nach 12 Monaten an die Ergebnisse der KG anglich. Die IG war mit dem Zeitpunkt des Beginns der Reha deutlich zufriedener als die KG. Alle anderen aus Fragebögen ermittelten Daten zeigten keine Unterschiede zwischen den beiden Gruppen.

**Schlussfolgerung:**

Der sehr frühe Beginn einer stationären Reha nach Cochleaimplantation ist erfolgreich umsetzbar. Die Reha konnte innerhalb von 7 Wochen nach der Implantation abgeschlossen werden. Der Vergleich der Ergebnisse der Tests des Sprachverstehens vor und nach der Reha zeigte eine deutliche Steigerung. Somit ist ein deutlicher Reha-Effekt nachweisbar. Die Aufnahme der CI-Rehabilitation in den Katalog der Anschlussheilbehandlungen ist somit wissenschaftlich begründet und damit dringend zu empfehlen.

Rehabilitation is an important part of cochlear implant (CI) treatment according to the German CI care guidelines updated in 2020. At present, the generally time-consuming approval process for the permission of inpatient rehabilitation delays the reintegration of patients affected by severe hearing impairment. In the present study, the feasibility of a very early inpatient rehabilitation program after CI surgery, designed as immediate rehabilitation (in German: *Anschlussheilbehandlung*, AHB), was examined. The data presented show that 92.6% of CI patients were able to start inpatient hearing rehabilitation within 14 days of their discharge from the hospital. Early rehabilitation after CI treatment (“Frankfurt concept”) was thus successfully evaluated as AHB.

## Basic and follow-up therapy after CI treatment

The cochlear implant (CI) is a neuroprosthesis that is used for patients with severe to profound hearing loss [15, 16]. The use of a CI can significantly improve the perception of speech [20], and the quality of life in everyday situations can be increased [18].

Once the indication has been established, implantation is followed by basic therapy. The basic therapy comprises the initial fitting phase of the CI audio processor with initial activation of the system and medical and audiological examinations. The subsequent follow-up therapy aims to achieve the best and fastest possible benefit from the implant system. Follow-up therapy consists of audiological therapy, hearing therapy, speech therapy, and medical therapy [9]. Once the follow-up therapy has been completed, lifelong aftercare is provided to ensure the best possible capacity to communicate.

## Contents and timing of a CI rehabilitation program

As part of the described CI treatment process, a rehabilitation program is recommended according to the 2020 updated guidelines for CI treatment [9]. The rehabilitation program can be part of both basic and follow-up therapy. Rehabilitation includes therapeutic content such as hearing and speech training, fitting of the processor, and counseling sessions. At the authors’ clinic, rehabilitation can take place on an inpatient basis in a rehabilitation clinic, usually over 3–5 weeks, or on an outpatient basis in an appropriate rehabilitation center. In this case, the outpatient appointments are spread over a period of several months. Patients are free to choose whether they wish to complete their rehabilitation on an outpatient or inpatient basis. In this study, only inpatient rehabilitation was considered, which was carried out at the MEDIAN Kaiserbergklinik Bad Nauheim.

Zeh and Baumann in 2015 demonstrated the effectiveness of an inpatient rehabilitation program on the perception of speech with CI. After completion of rehabilitation, the results of various tests of speech perception showed an average improvement of around 20 percentage points [22].

To date, there is no standardized concept in Germany that describes the rehabilitation process after CI treatment. The structural implementation of basic and follow-up therapy is usually based on individual, local concepts of the respective CI providing institution. Rehabilitation can be carried out on an inpatient, outpatient, or mixed basis. An individual assessment and approval by the cost bearer is currently required for each individual case. At present, an application for rehabilitation (application form number *G100*) must be submitted to the responsible cost bearer (health insurance, pension insurance). The rejection rate for adults varies depending on the German federal state. In individual cases, CI patients had to fight for their right to inpatient rehabilitation in the social courts [4]. This leads to a considerable delay in the start of rehabilitation.

In contrast to CI rehabilitation, inpatient rehabilitation is initiated in Germany immediately after many other surgeries or diseases, such as after the implantation of endoprostheses or bypass surgeries. This type of rehabilitation is referred to as “immediate rehabilitation” (in German: *Anschlussheilbehandlung*, AHB). The start of rehabilitation within 14 days of discharge from hospital is an essential prerequisite for the initiation of AHB [8]. For CI treatment, this raises the question of whether rehabilitation is also possible very early after implantation, ideally within 14 days of discharge from hospital. This would fulfill the formal requirements for CI rehabilitation as AHB (*Anschlussheilbehandlung*).

To date, the initial fitting of the CI audio processor was generally carried out after a healing phase of around 3–6 weeks, meaning that rehabilitation could not even begin within the first 2 weeks of discharge from hospital [12, 15]. Thanks to improved surgical techniques, such as the small-incision technique [17], it is now feasible to activate the CI audio processor as early as 2–3 days after implantation [1]. Previous studies have already shown that early activation of the CI audio processor can be successfully implemented within a few days of implantation and that it leads to equivalent hearing success to an initial fitting after the standard healing phase [5, 6, 11]. Fitting the CI audio processor immediately after the surgery also makes it possible to start rehabilitation very early after CI implantation. An important advantage of early rehabilitation is that the patient can be reintegrated into work and everyday life much more quickly. So far, however, it is not possible to start CI rehabilitation in Germany without the approval of the responsible cost bearer.

## Aim of the study

The aim of the present study was therefore to investigate the feasibility of starting early inpatient rehabilitation within 14 days (maximum 28) of CI implantation. The results were compared with the data from a control group in which rehabilitation is carried out according to the previous standard process. This pilot project was made possible by cooperation between the ENT University Clinic Frankfurt, the MEDIAN Kaiserberg-Klinik Bad Nauheim, and various cost bearers: German Pension Insurance (*Deutsche Rentenversicherung*, DRV) Bund, DRV Hessen, Knappschaft Bahn/See, *Deutsche Angestellten-Krankenkasse* (DAK). This has considerably simplified the application process for CI rehabilitation and significantly shortened the waiting time for admission to the rehabilitation clinic.

The main question of the study was whether patients with very short CI experience can benefit from a rehabilitation program in the same way as patients after several months of CI usage and thus longer habituation.

## Material and method

### Patients

Two groups were formed to investigate the research question (intervention group and control group). In total, 54 patients (23 male, 31 female) were included in the intervention group (IG). In this group, the (inpatient) rehabilitation program was started very early (target within 14 days, maximum 28 days) after CI surgery. In the control group (CG), 21 patients (6 male, 15 female) were included. The rehabilitation was applied postoperatively according to the usual process via an application for rehabilitation at the responsible cost bearer. Further inclusion criteria were a minimum age of 18 years and unilateral or bilateral CI treatment. All potential CI candidates could be included in the patient population, including patients with single-sided deafness (SSD). The demographic data of the patient groups are shown in Table 1. All study participants underwent an early fitting of the CI audio processor within 3 days of CI surgery [5, 11]. Regardless of assignment, both groups underwent the previously described inpatient rehabilitation program for hearing rehabilitation with CI [22].

**Table 1:** Demographic data of the intervention group and the control group

	Intervention group	Control group
Age (mean value)	51.7 years	52.9 years
Minimum/maximum	19/86 years	43/65 years
*Type of treatment*		
Bilateral	15	9
Bilateral single-stage	4	–
Bimodal	20	10
Unilateral	15	2
Duration of hearing loss (mean value)	21.8 years	27.3 years
Minimum/maximum	1/58 years	5/50 years
Hearing aid experience (mean value)	20.9 years	22.8 years
Minimum/maximum	1/50 years	2/46 years
*Implants*		
HiFocus™ 3D Ultra SlimJ^a^	3	–
HiFocus™ 3D Ultra Mid-Scale^a^	1	–
CI512^b^	–	2
CI612^b^	17	10
CI622^b^	2	–
CI632^b^	6	2
Flex24^c^	–	1
Flex26^c^	10	3
Flex28^c^	14	3
FlexSoft^c^	1	–
*Processors*		
CP1000^b^	18	6
CP1150^b^	5	3
CP950^b^	2	5
Naída CI M90^a^	2	–
Naída CI Q90^a^	2	–
Rondo 2^c^	1	–
Rondo 3^c^	11	1
Sonnet 2^c^	13	6

^a^ Advanced Bionics, Valencia (CA), USA

^b^ Cochlear, Macquarie, Australia

^c^ MED-EL, Innsbruck, Austria

### CI treatment process

The CI treatment process at the authors’ clinic is based on the recommendations of the CI guideline [3, 9] and included the following sub-areas within the study:

- Indication
- Implantation
- Basic therapy
- Follow-up therapy

The audiological-medical part of the basic therapy covered a maximum period of 2 weeks after the CI surgery. It included the initial fitting phase of the processor as well as wound examination and was carried out over three appointments at the hospital. The first appointment took place 2–3 days after the implantation (day of discharge from hospital) and included the wound examination and, after the patient was able, the initial activation of the processor. At two further outpatient appointments, the wound healing was medically examined, the processor fitting was further refined (basic audiological therapy), and audiological diagnostics were carried out to assess the hearing benefit. As part of the study, basic and follow-up therapy for hearing and speech perception was provided as immediate rehabilitation (AHB) at the cooperating rehabilitation institution. The outsourcing of hearing and speech therapy to an external rehabilitation institution complies with the requirements of the current German CI guideline [3] and the German *Weißbuch CI-Versorgung* [9], as this is a part of the CI treatment process that can be delegated by the CI-providing clinic. The rehabilitation was carried out externally as inpatient rehabilitation at the Kaiserbergklinik Bad Nauheim. The number of rehabilitation days approved by the cost bearer were completed consecutively in the rehabilitation institution. Therapeutic measures were carried out during rehabilitation, including hearing training, fittings of the audio processor, counseling, individual therapy and group therapy. As part of the follow-up therapy, outpatient appointments were held at the institution providing the CI (ENT clinic) 3, 6, and 12 months after the CI surgery. The medical and audiological follow-up therapy was carried out here. In order to compare the early rehabilitation with the standard beginning, the rehabilitation period of both groups was recorded. For this, we documented the waiting time between discharge from hospital and the start of rehabilitation as well as the duration of inpatient rehabilitation.

In order to compare the therapy results of both study groups, the speech perception with the CI was determined. For this purpose, speech perception in quiet was measured using the Freiburg monosyllabic test [13] at 65 dB SPL in free field. The measurement was performed monaurally; with usable residual hearing in the contralateral ear, plug-in earphones were used for masking (broadband noise, level 70 dB HL). In addition, speech perception in noise was measured using either the HSM (Hochmair–Schulz–Moser) sentence test [14] at a speech level of 65 dB SPL and a signal-to-noise ratio of +10 dB (test condition in the rehabilitation clinic), or the Oldenburg sentence test (OlSa) [21] was performed at a fixed speech level of 65 dB SPL and adaptive noise level (test condition in the ENT university clinic). Both measurements in noise were carried out monaurally in free field with speech and noise signal from the front (S_0_N_0_ condition). If there was usable residual hearing, the contralateral ear was double-blocked with an earplug and hearing protection during the OlSa tests (Peltor, 3M, Neuss, Germany). To evaluate the subjective hearing perception of the patients, the Speech, Spatial and Qualities of Hearing Scale (SSQ) questionnaire [10] and the Hearing Implant Sound Quality Index (HISQUI) questionnaire [2] were administered. A self-developed questionnaire was used to record the patients’ subjective satisfaction with regard to the time of the start of rehabilitation. Here, a Likert scale with five points (*very satisfied, satisfied, moderately satisfied, rather dissatisfied, not satisfied*) was applied. With the help of a questionnaire to be completed by the audiological staff, the fitting effort was assessed using a three-point Likert scale (*short, medium, long effort*) and the average processor usage time was documented using the data logging function of the corresponding CI fitting software.

**Fig. 1:**
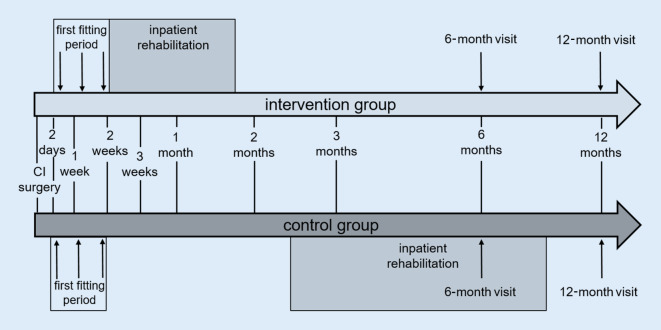
Schematic illustration of the study process over time (*top *intervention group, *bottom *control group)

Data were collected preoperatively (pre-op), at the first fitting (FF) of the CI audio processor, on admission and discharge from rehabilitation, and at the regular clinical follow-up appointments at 6 and 12 months (6M/12M) after CI surgery. The study procedure is shown schematically in Fig. 1.

### Statistical analysis

After prior verification of normal distribution (Kolmogorov–Smirnov test, *p* > 0.05), the data were analyzed for significant differences using parametric tests such as paired *t *test (one-sided) or two-factorial repeated-measures (RM) analysis of variance (ANOVA) with Bonferroni correction for multiple comparisons. If there was no normal distribution, the Mann–Whitney *U* test (MWU) was used for comparisons between the groups, and the Wilcoxon rank sum test (WRS) was used for comparisons within the groups. A significance level of *p* *=* 0.05 was set for the statistical analysis. The data analysis was carried out using SPSS Statistics 24.0 (IBM Corporation, Endicott, NY, USA). All numerical values reported in the results section are central values (medians).

## Results

### Rehabilitation period

**Fig. 2:**
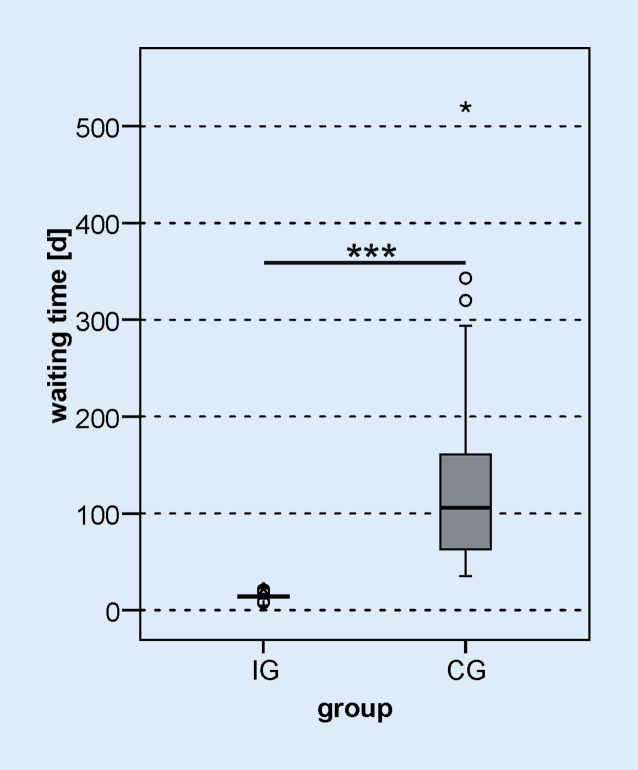
*Boxplot *of waiting time between CI surgery and start of inpatient rehabilitation. *d* days, *IG* intervention group, *CG* control group; *** *p* < 0.001

**Fig. 3:**
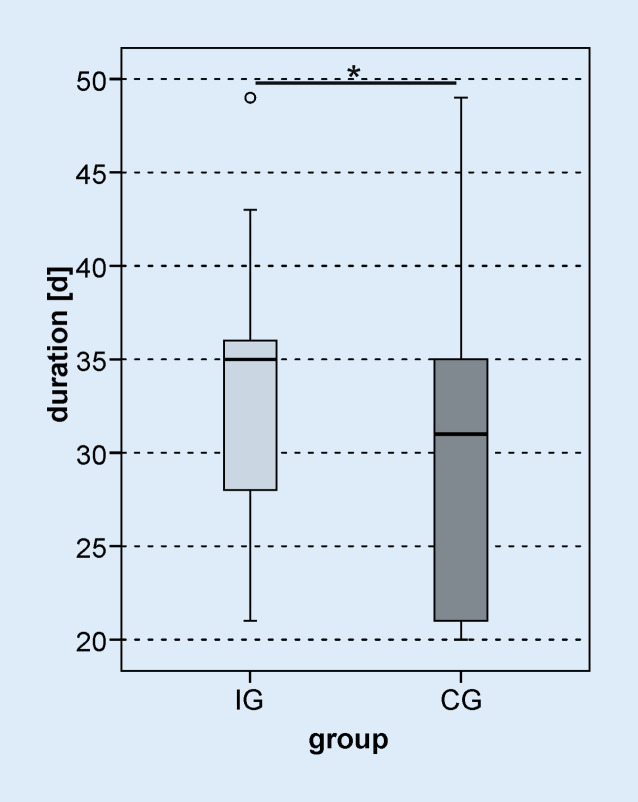
*Boxplot *of the duration of inpatient rehabilitation. *d* days, *light gray* intervention group (*IG*), *dark gray *control group (*CG*); * *p* < 0.05

The waiting time between CI surgery and the start of inpatient rehabilitation was significantly shorter in the IG than in the CG (*z*_*MWU*_ *=* −6.827; *p*_*MWU*_ < 0.001, Fig. 2). In the IG, the waiting time was 14 days (minimum 8 days, maximum 23 days), while the CG was able to start rehabilitation after 106 days (minimum 35 days, maximum 520 days). For two patients in the IG, the waiting time for rehabilitation was 8 days. Both patients completed all three initial fitting appointments in a short time and thus completed the audiological basic therapy in the clinic. Overall, 92.6% of the IG patients were able to start rehabilitation within 14 days. There was also a significant difference in the duration of inpatient rehabilitation (Fig. 3) between the study groups (IG: 35 days, CG: 31 days, *z*_*MWU*_ = −2.226; *p*_*MWU*_ = 0.026). The IG was able to complete rehabilitation 49 days (minimum 29, maximum 63) after CI treatment; the CG, on the other hand, only completed rehabilitation after 129 days (minimum 69, maximum 555).

### Speech perception in quiet

**Fig. 4:**
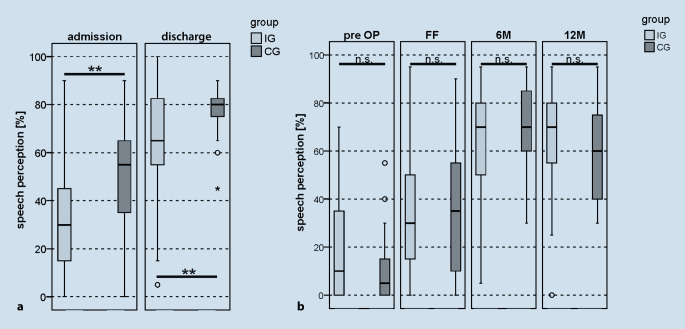
*Boxplot *of the results for speech perception (%) in quiet, Freiburg monosyllabic test (65 dB SPL, free field). **a** On admission and discharge from inpatient rehabilitation, and **b** at the regular appointments in the clinic: *pre-op *Preoperative, *FF *after completion of the first fitting phase of the processor, *6M* 6-month follow-up, *12M* 12-month follow-up. *Light gray *intervention group (*IG*), *dark gray *control group (*CG*); *n.* *s. *not significant, ** *p* < 0.01

Both groups benefited from CI treatment and showed significantly improved monosyllabic speech perception postoperatively compared to the preoperative results (IG *z*_*WRS*_ = −3.799; *p*_*WRS*_ < 0.001; CG _*TT_paired*_ = −6.183; _*pT_paired*_ < 0.001). The effect of rehabilitation determined by comparing the results at admission and discharge showed that the IG benefited more (35 percentage points) than the CG, which showed an improvement of 25 percentage points. However, this difference in the improvement in speech perception is not statistically significant (_*TT_unpaired*_ = 1.386; _*pT_unpaired*_ = 0.170). The monosyllabic speech perception assessed preoperatively and at the 6M/12M intervals showed no significant difference between the two groups (IG/CG 6M 70%/70%, _*TT_unpaired*_ = −0.716; _*pT_unpaired*_ = 0.477; 12M 70%/60%, _*TT_unpaired*_ = 0.731; _*pT_unpaired*_ = 0.469; Fig. 4b). The CG showed significantly better monosyllabic speech perception compared to the IG both at admission and at discharge from rehabilitation (IG/CG admission 30%/55%, _*TT_unpaired*_ = −3.075; _*pT_unpaired*_ _*=*_ 0.003; discharge 65%/80%, _*TT_unpaired*_ = −2.832; _*pT_unpaired*_ = 0.006; Fig. 4a).

### Speech perception in noise

**Fig. 5:**
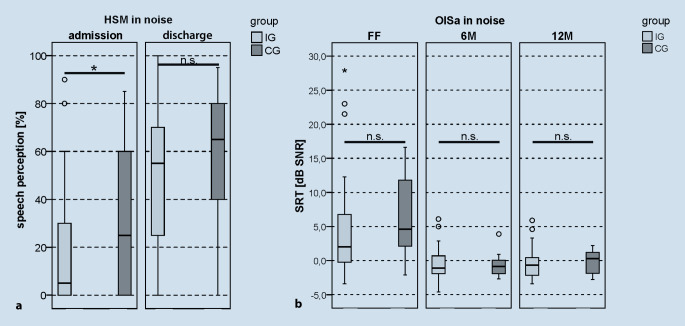
*Boxplot *of the results for speech perception in noise. **a** Speech perception (%), HSM in noise on admission and discharge from inpatient rehabilitation. **b** Speech recognition threshold (SRT*; *dB SNR), OlSa in noise after completion of the first fitting phase (*FF*), at the 6‑month follow-up (*6M*) and at the 12-month follow-up (*12M*). *Light gray *intervention group (*IG*), *dark gray *control group (*CG*), *HSM *Hochmair–Schulz–Moser sentence test, *OlSa *Oldenburg sentence test; *n.* *s. *not significant, *p < 0.05

Both groups showed a significant improvement in the speech perception in noise when comparing the results between admission and discharge from rehabilitation (admission/discharge: IG 5%/55%, *z*_*WRS*_ = −5.506; *p*_*WRS*_ < 0.001; CG 25%/65%, _*TT_paired*_ = −4.558; _*pT_paired*_ < 0.001; Fig. 5a). As with the test of speech perception in quiet, the IG achieved a 10 percentage point greater benefit after completion of rehabilitation. However, this was not statistically significant (_*TT_unpaired*_ = 0.971; _*pT_unpaired*_ = 0.335). At the 6M interval, both the IG and the CG showed a significant improvement in the OlSa-SRT results compared to the measurement at initial fitting of the CI audio processor (FF/6M: IG: 2.0 dB SNR/−1.1 dB SNR; CG 4.6 dB SNR/−0.85 dB SNR; Fig. 5b). In the 12M interval, the results in both groups remained stable compared to the 6M results (6M/12M: IG −1.1 dB SNR/−0.65 dB SNR; *z*_*WRS*_ = −0.278; *p*_*WRS*_ = 0.781; CG −0.85 dB SNR/0.3 dB SNR; _*TT_paired*_ = 2.187; _*pT_paired*_ = 0.094).

A group comparison showed significantly better results in the HSM sentence test in noise for the CG on admission to rehabilitation (CG: 25%, IG: 5%, *z*_*MWU*_ = −2.035; *p*_MWU_ = 0.042), which converged on discharge (IG: 65%, CG: 55%, _*TT_unpaired*_ = −1.327; _*pT_unpaired*_ = 0.189).

### Subjective hearing perception

**Fig. 6:**
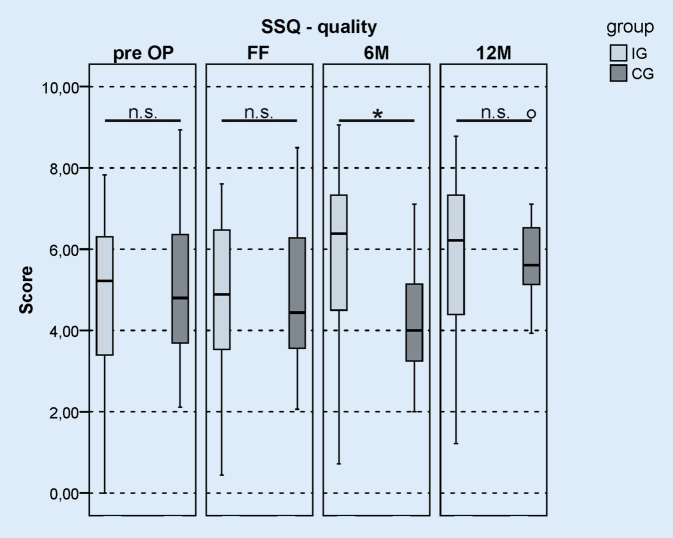
*Boxplot *of the results of the Speech, Spatial and Qualities of Hearing Scale (SSQ) questionnaire, subjective assessment of hearing quality. Time: preoperative (*pre-op*), after completion of the first fitting (*FF*), at the 6‑month follow-up (*6M*), and at the 12-month follow-up (*12M*). *Light gray *intervention group (IG), *dark gray *control group (CG); *n.* *s. *not significant, *p < 0.05

The SSQ questionnaire records the subjective hearing perception in three sub-areas: speech perception, spatial hearing, and hearing quality. The results of the questionnaire for the three sub-areas are shown in Table 2. For hearing quality, there was a significant difference between IG and CG at the 6M interval, whereby the hearing quality was rated better in the IG than in the CG (Fig. 6). After 12 months of CI usage, there was no significant difference between the groups. In the areas of speech perception and spatial hearing, there was no significant difference between the groups at any time (Table 2). There was a tendency for the IG patients to give slightly more favorable ratings than their CG counterparts at the 6M interval. The results of the HISQUI questionnaire showed no significant difference between IG and CG at all observation times (Table 3). The results of the HISQUI questionnaire also showed a tendency for the IG to give a higher rating of hearing quality than the CG at the 6M interval.

**Table 2:** Results of the SSQ for the sub-areas hearing quality, speech perception, and spatial hearing at various observation times

**Hearing quality**				
Time	Score IG	Score CG	*T*_*T_unpaired*_	*p*_*T_unpaired*_
Pre-op	5.2	4.8	−0.723	0.472
FF	4.9	4.4	0.236	0.814
6M	6.4	4.0	2.040	**0.047**
12M	6.2	5.6	−0.015	0.988
**Speech perception**				
Time	Score IG	Score CG	*T*_*T_unpaired*_	*p*_*T_unpaired*_
Pre-op	3.7	3.4	−4.11	0.683
FF	4.6	3.6	0.851	0.399
6M	4.9	4.0	1.626	0.111
12M	5.4	4.6	0.479	0.635
**Spatial hearing**				
Time	Score IG	Score CG	*T*_*T_unpaired*_	*p*_*T_unpaired*_
Pre-op	2.4	3.5	−1.634	0.108
FF	3.9	3.8	−0.285	0.776
6M	5.1	3.9	0.816	0.419
12M	5.3	5.2	−0.125	0.901

*SSQ *Speech, Spatial and Quality of Hearing Scale, *Pre-op* preoperative, *FF* after completed first fitting phase, *6M* at 6‑month follow-up-, *12M* 12-month follow-up, *IG* intervention group, *CG* control group

Statistical values: *T* value (*T*_*T_unpaired*_) and *p *value (*p*_*T_unpaired*_) after *t *test for unpaired samples

**Table 3:** Results of the HISQUI questionnaire at various observation times

Time	Points IG	Points CG	*T*_*T_unpaired*_	*p*_*T_unpaired*_
Pre-op	69.0	59.5	−0.128	0.889
FF	71.0	71.0	1.006	0.319
6M	81.0	65.0	1.128	0.265
12M	81.0	82.0	0.121	0.904

*HISQUI *Hearing Implant Sound Quality Index, *Pre-op* preoperative, *FF* after completed first fitting phase, *6M* 6-month follow-up, *12M* 12-month follow-up, *IG* intervention group, *CG* control group

Statistical values: *T* value (*T*_*T_unpaired*_) and *p *value (*p*_*T_unpaired*_) after *t* test for unpaired samples

### Audiological questionnaire

The examination of the time required for the fitting of the CI audio processor showed no significant difference between the study groups (FF: *z*_*MWU*_ = −0.517; *p*_*MWU*_ = 0.605; 6M: *z*_*MWU*_ = −0.563; *p*_*MWU*_ = 0.573; 12M: *z*_*MWU*_ = −0.213; *p*_*MWU*_ = 0.831). At all appointments, the majority of patients (IG: 58.6–78.8%, CG: 50–92.3%) required a medium fitting effort. The results also showed a comparable mean processor usage time between the IG (FF: 11.9 h/day, 6M: 13.5 h/day, 12M: 13.0 h/day) and CG (FF: 10.3 h/day, 6M: 13.8 h/day, 12M: 13.7 h/day) at all study time points (FF: _*zT_unpaired*_ = 1.388; _*pT_unpaired*_ = 0.178; 6M: *z*_*MWU*_ = −0.641; *p*_*MWU*_ = 0.522; 12M: _*TT_unpaired*_ = −0.167; _*pT_unpaired*_ = 0.868).

### Subjective satisfaction with the time of rehabilitation

The patients in the IG showed a very high level of subjective satisfaction with the time of the early rehabilitation at the 12M interval. Here, 88.5% were “very satisfied” and 11.5% “satisfied” with the time of the start of rehabilitation. The assessments of the start of rehabilitation in the CG were significantly worse than in the IG (*z*_*MWU*_ = −2.583; *p*_*MWU*_ = 0.01). In this group, only 50% were “very satisfied,” 30% were satisfied, 10% were moderately satisfied, and 10% were dissatisfied.

## Discussion

The results of the study showed that very early rehabilitation after CI surgery can be carried out successfully. The results did not reveal any disadvantages compared to the usual rehabilitation process. Patients with very short CI hearing experience benefit from the rehabilitation program in the same way as patients after several months of CI use.

### Time of rehabilitation

By starting basic therapy just 2 days after implantation, in conjunction with the acceleration of the application and approval process, the waiting time for the start of inpatient rehabilitation was reduced to around 2 weeks. In the IG, 92.6% of patients were able to start rehabilitation within 14 days. The remaining 7.4% of patients started rehabilitation within 23 days. Reasons for the later start of rehabilitation included the occurrence of postoperative swelling, which required a pressure bandage for several days. This led to a postponement of the initial fitting by a few days and thus also to a delay in the start of rehabilitation. In other cases, rehabilitation was not started within 14 days of CI treatment for personal or organizational reasons (e.g., public holidays). In the IG, rehabilitation was completed on average just 7 weeks after CI treatment, while most patients in the CG were still waiting for rehabilitation to begin at this time. Despite the very early start of rehabilitation, the IG only needed slightly longer on average (4 days) to complete the rehabilitation program.

### Speech perception results

The two study groups showed a significant improvement in speech perception both in quiet and in noise after completion of rehabilitation. The improvement in speech perception from the beginning to the end of rehabilitation was 10 percentage points (mean value) greater in the IG than in the CG. This could be due to the fact that the CG showed significantly better test results at admission than the IG and therefore a slightly smaller benefit was achieved through rehabilitation.

When interpreting the results of admission and discharge, it should be noted that the time point of the assessment differs significantly between the IG and CG. The IG achieved better results 7 weeks after implantation and completion of rehabilitation, both at quiet and with background noise, than the CG after 15 weeks (at the start of rehabilitation). This is relevant for situations in everyday life. The results thus prove that the Frankfurt concept provides CI patients with the benefit of the CI significantly earlier and thus achieves an earlier improvement in speech perception.

Both on admission and at discharge from the rehabilitation program, the CG showed better monosyllabic speech perception than the IG. When interpreting the results, it should be noted that patients in the CG already had significantly more hearing experience with the CI than patients in the IG at this time. While patients in the IG had only been able to use the CI audio processor for a few days before the start of rehabilitation, those in the CG had already been able to gain hearing experience for several weeks or even months before rehabilitation. Both study groups showed a sufficient and comparable average duration of processor use over time [7]. Consistent use of the CI in everyday life therefore appears to lead to an improvement in speech perception even without a structured rehabilitation program. However, rehabilitation leads to a significant improvement of speech perception with the CI. The present results are therefore consistent with earlier studies that were also able to demonstrate the positive effect of rehabilitation on the benefits of CI [22].

The results for the 6M and 12M interval showed a comparable development in both groups for speech test results in quiet and in noise after the inpatient rehabilitation program was completed. No significant differences were found between the two groups at any time. Thus, the data collected by the authors show a comparable effect of the rehabilitation program even at a very early start.

For 92.6% of patients, the rehabilitation program could start within 14 days of surgery. The comparison of the IG with the CG showed no significant differences in speech perception in quiet and in noise, neither at admission and discharge from rehabilitation nor after 6 months of CI use. Accordingly, comparable speech test results can be achieved if rehabilitation starts within 14 days of CI treatment.

### Results of the SSQ questionnaire

The results of the SSQ questionnaire at the 6M interval showed significantly better ratings in the IG than in the CG in the area of hearing quality. The IG therefore appeared to benefit subjectively earlier from rehabilitation than the CG. After 6 months, 40% of the patients in the CG had not yet completed rehabilitation. The results of the SSQ questionnaire in the areas of speech perception and spatial hearing as well as the data from the HISQUI questionnaire did not show a significant difference between the groups at any point in the study. However, there was a tendency for the IG to make slightly higher judgments at the 6M interval compared with the CG.

### Subjective satisfaction with the time of rehabilitation

The very early initiation of rehabilitation was rated very positively by the patients. All patients in the IG showed a high degree of satisfaction with the timing of the start of rehabilitation. By contrast, there were some patients in the CG who were only moderately satisfied or rather dissatisfied with the start of rehabilitation. This is presumably due to the sometimes very long waiting times of up to almost 1 year between implantation and rehabilitation.

### Use of a single-unit processor

When the processor was first activated, a single-unit processor worn directly over the implant was used for a total of 27 patients (33% of the IG and 43% of the CG). In 22 cases there were no problems with the initial activation. Mostly a stronger magnet was used, which could be weakened in the subsequent technical control sessions. In two cases (one IG and one CG) the magnet force was very weak, but with the use of a headband it was possible to wear the single-unit processor. In two other cases (both IG), the magnetic force of the single-unit processor was too weak on initial activation, so that a temporary switch was made to a behind-the-ear device (BTE). At the end of the basic therapy, it was then possible to switch back to the single-unit processor. In one case (CG), sufficient retention was not achieved with the single-unit processor, so that a permanent switch to a BTE device had to be made. There was no difference in the applicability of single-unit processors between the groups. Early basic therapy and early rehabilitation therefore do not appear to require the use of BTE processors to a greater extent.

### Organizational concept of rehabilitation

The concept of inpatient rehabilitation of CI patients pursued in this study has already been presented in form and content in a previous publication [22]. In addition to this organizational concept of rehabilitation, other alternative concepts exist in Germany. In particular, outpatient rehabilitation of CI users is being pursued in many places. Presumably, the model of a very early start to rehabilitation can also be transferred to other concepts. However, a comparison of the concepts was not the aim of this study.

### CI rehabilitation as AHB

In the present study, it was shown that more than 90% of the patients in the intervention group started a rehabilitation program within 14 days of discharge. This proved that the vast majority of patients fulfilled the formal criteria for postoperative rehabilitation (follow-up therapy) as AHB. Respecting the tight time constraints of AHB requires the fulfillment of important conditions. Firstly, basic therapy must have been completed at the implanting clinic before the start of inpatient rehabilitation. The updated German CI guideline requires a medical examination and audiological basic therapy as part of the activation of the CI system in the CI-providing hospital (in German: *CI-versorgende Einrichtung*, CIVE; [3]). This process flow is therefore also described in the specifications for CIVE certification [19].

For this reason, basic therapy as a key component of the CI treatment process cannot be delegated and must be carried out at the implanting facility. The activation of an active implanted neuroprosthesis is the medical responsibility of the “operator” of the medical device (according to the German Medical Device Directive, *Medizinprodukte-Betreiberverordnung*). Therefore, a physician must first assess whether the patient fulfills the medical, audiological, and psychological requirements to be able to start postoperative basic therapy. A number of factors or unexpected events can not only compromise the start of basic therapy, but can even endanger the patient’s health. The conditions must therefore be continuously assessed before and during basic therapy, as they can change significantly and immediately. Examples include the medical assessment of the wound with regard to wound infections, swelling of the wound, swelling of the coil region and skin thickness, assessment of the pressure force of the transmitting coil magnet, possible pressure marks or skin necrosis, and pain sensations. In addition, the patient’s psychological situation after the surgery must be assessed. Only after an individual medical assessment can the patient’s condition be approved by a physician for audiological basic therapy. As part of the audiological basic therapy, the basic function of the implant system has to be ensured, including fitting of the CI processor.

The start of basic therapy should therefore by no means be taken for granted, but requires individual medical assessment as part of a guideline-based treatment process [3, 9], especially with regard to the timing of the start of basic therapy. Due to the implementation of the concept of early activation of the CI system [5, 11], the start of basic therapy can now be significantly accelerated. In many cases, basic therapy can be performed successfully in the first few days after surgical implantation. Even if this concept is applicable to the majority of patients, it does not apply to all patients. However, early activation of the CI system is the requirement for fulfilling the predefined AHB time limit of 14 days. It is therefore obvious that the majority of patients, but not all, are suitable for using the AHB procedure as an admission to immediate rehabilitation. The assessment of postoperative “rehabilitation suitability” is therefore an important, non-delegable task of the CIVE, which makes a central contribution to the quality assurance of CI treatment. Even if AHB can be regarded as the best solution for the majority of patients, structures should also be developed in future for the remaining patients to enable an unbureaucratic access to rehabilitation outside the AHB procedure.

### Rating of the study

The results of this pilot study showed that CI patients benefit from very early inpatient rehabilitation (Frankfurt concept). With earlier rehabilitation, patients can be reintegrated into work and everyday life much sooner. Furthermore, the simplified AHB procedure considerably reduces the bureaucratic workload for all parties involved. There were no limitations associated with the early start of rehabilitation, particularly with regard to the effect of the rehabilitation program.

### Limitations of the study

The aim of this study was to demonstrate the feasibility and effectiveness of very early inpatient CI rehabilitation in order to draw conclusions about the application of the AHB procedure. A motivation-related bias cannot be ruled out here, as the assignment to the study group was made by the patients themselves, in that they decided for or against very early inpatient rehabilitation in analogy to an AHB procedure. In addition, due to the COVID-19 pandemic, only a few individuals could be included in the control group. For the same reason, some patients were unable to attend all regular clinical follow-up appointments; in addition, some patients were unable to participate for personal reasons. The results presented here were obtained for both the IG and the CG as part of an inpatient rehabilitation program. It is therefore not possible to assess whether comparable results can be achieved with alternative, e.g., outpatient rehabilitation concepts.

## Outlook

The results of this pilot study suggest that CI rehabilitation according to the Frankfurt concept should be included in the catalog of immediate rehabilitation (AHB). At present, AHB is defined as a medical rehabilitation service that is provided immediately (within 14 days) after inpatient hospital treatment. It can be started in the fast or direct initiation procedure [8]. The inclusion of CI rehabilitation in the AHB catalog would have enormous advantages for patients, physicians, rehabilitation clinics, and cost bearers alike. As an AHB program, the application process would be significantly shortened and greatly simplified administratively. In addition, every patient would immediately be entitled to rehabilitation after CI surgery, as long as this was started within 2 weeks of discharge. By including CI rehabilitation into the AHB catalog, the CI guideline-based treatment process would be implemented consistently throughout Germany. Patients affected by severe hearing impairment or deafness would thus achieve a much faster improvement in their hearing performance and speech perception, which in turn could significantly accelerate their professional and social reintegration. The latter aspect in particular is also in the direct interest of the cost bearers. The results obtained with the Frankfurt concept thus make an important scientific contribution to the re-evaluation of rehabilitation after CI treatment as immediate rehabilitation (AHB).

## Practical conclusion

- Cochlear implant (CI) treatment includes basic and follow-up therapy.
- Basic therapy comprises medical examinations, the start of audiological therapy, including initial activation of the processor, and hearing and speech therapy.
- Subsequent follow-up therapy continues with the aforementioned components, and focuses on hearing therapy and audiological follow-up therapy.
- Parts of basic and follow-up therapy can also be performed as inpatient rehabilitation.
- Rehabilitation enables intensive and individualized hearing training to improve hearing performance with the CI.
- Starting inpatient rehabilitation within 2 weeks of CI implantation can be successfully implemented.
- The positive effect of inpatient rehabilitation can also be seen when rehabilitation begins very early.
- Completion of rehabilitation after about 7 weeks with a strong improvement in hearing ability was shown.
- Including CI rehabilitation in the German catalog of immediate rehabilitation (AHB) is scientifically justified and thus highly recommended.
- The implementation of CI-AHB would guarantee a standardized quality of treatment for all patients throughout Germany.

## Declarations

All described investigations on humans or human tissue were conducted in accordance with national law and the Declaration of Helsinki of 1975 (in the current, revised version). The study was approved by the Ethics Committee of the Faculty of Medicine of the J. W. Goethe University Frankfurt am Main (case number 19-3089). A declaration of consent was obtained from all participating patients.

The supplement containing this article is not sponsored by industry.

## References

[1] Alsabellha RM, Hagr A, Al-Momani MO, Garadat SN (2014) Cochlear implant device activation and programming: 5 days postimplantation. Otol Neurotol 35(4):e130–e134. 10.1097/MAO.0000000000000266 24622029 10.1097/MAO.0000000000000266

[2] Amann E, Anderson I (2014) Development and validation of a questionnaire for hearing implant users to self-assess their auditory abilities in everyday communication situations: the Hearing Implant Sound Quality Index (HISQUI19). Acta Otolaryngol 134(9):915–923. 10.3109/00016489.2014.909604 24975453 10.3109/00016489.2014.909604

[3] AWMF S2k-Leitlinie Cochlea-Implantat Versorgung. AWMF-Register-No. 017/071 (S2k- guidelines for the supply of cochlear implant)

[4] Baumann U (2019) Cochlea-Implantat-Träger erstreitet Bewilligung einer stationären Hör-Rehabilitation. Das interessante Urteil. Z Audiol 58(1):24–25

[5] Bruschke S, Baumann U, Stöver T (2021) Long-term follow-up of early cochlear implant device activation. Audiol Neurootol 26(5):327–337. 10.1159/000512760 33657558 10.1159/000512760

[6] Bruschke S, Baumann U, Stöver T (2023) Residual low-frequency hearing after early device activation in cochlear implantation. Eur Arch Otorhinolaryngol. 10.1007/s00405-023-07887-0 36943438 10.1007/s00405-023-07887-0 PMC10382339

[7] Craddock L, Cooper H, Riley A, Wright T (2016) Cochlear implants for pre-lingually profoundly deaf adults. Cochlear Implants Int 17(Suppl 1):26–30. 10.1080/14670100.2016.1161122 27099107 10.1080/14670100.2016.1161122

[8] Deutsche Rentenversicherung Bund (2017) Medizinische Voraussetzungen der Anschlussrehabilitation (AHB). AHB-Indikationskatalog

[9] DGHNO-KHC (2021) Weißbuch Cochlea-Implantat(CI)-Versorgung, 2 edn.

[10] Gatehouse S, Noble W (2004) The speech, spatial and qualities of hearing scale (SSQ). Int J Audiol 43(2):85–99. 10.1080/14992020400050014 15035561 10.1080/14992020400050014 PMC5593096

[11] Günther S, Baumann U, Stöver T (2018) Early fitting in cochlear implantation. Benefits and limits. Otol Neurotol 39(4):e250–e256. 10.1097/MAO.0000000000001745 29533333 10.1097/MAO.0000000000001745

[12] Hagr A, Garadat SN, Al-Momani M, Alsabellha RM, Almuhawas FA (2015) Feasibility of one-day activation in cochlear implant recipients. Int J Audiol 54(5):323–328. 10.3109/14992027.2014.996824 25634774 10.3109/14992027.2014.996824

[13] Hahlbrock K‑H (1953) Über Sprachaudiometrie und neue Wörterteste. Arch Ohren Nasen Kehlkopfheilkd 162(5):394–431. 10.1007/BF02105664 13092895

[14] Hochmair-Desoyer I, Schulz E, Moser L, Schmidt M (1997) The HSM sentence test as a tool for evaluating the speech understanding in noise of cochlear implant users. Otol Neurotol 18:S83–S86 9391610

[15] Hoppe U, Liebscher T, Hornung J (2017) Anpassung von Cochleaimplantatsystemen. HNO 65:546–551. 10.1007/s00106-016-0226-7 27538936 10.1007/s00106-016-0226-7

[16] Lenarz T (2017) Cochlear implant—State of the art. Laryngorhinootolologie 96(S01):S123–S151. 10.1055/s-0043-101812 10.1055/s-0043-101812 28499298

[17] Prager JD, Neidich MJ, Perkins JN, Meinzen-Derr J, Greinwald JH (2012) Minimal access and standard cochlear implantation. A comparative study. Int J Pediatr Otorhinolaryngol 76(8):1102–1106. 10.1016/j.ijporl.2012.04.008 22595461 10.1016/j.ijporl.2012.04.008

[18] Rader T, Haerterich M, Ernst BP, Stöver T, Strieth S (2018) Lebensqualität und Schwindel bei bilateraler Cochleaimplantation. Fragebogeninstrumente zur Qualitätssicherung. HNO 66(3):219–228. 10.1007/s00106-017-0456-3 29230508 10.1007/s00106-017-0456-3

[19] Stöver T, Plontke SK, Guntinas-Lichius O, Welkoborsky H‑J, Zahnert T, Delank KW, Deitmer T, Esser D, Dietz A, Wienke A, Loth A, Dazert S (2023) Konzeption und Implementierung eines Zertifizierungssystems zur Qualitätssicherung der Cochlea-Implantat-Versorgung in Deutschland. HNO 71(6):396–407. 10.1007/s00106-023-01305-x 37115246 10.1007/s00106-023-01305-x PMC10234877

[20] Sun C‑H, Chang C‑J, Hsu C‑J, Wu H‑P (2019) Feasibility of early activation after cochlear implantation. Clin Otolaryngol. 10.1111/coa.13427 31487432 10.1111/coa.13427

[21] Wagener K, Kühnel V, Kollmeier B (1999) Entwicklung und Evaluation eines Satztests für die deutsche Sprache. I: Design des Oldenburger Satztests. Z Audiol 38:4–15

[22] Zeh R, Baumann U (2015) Stationäre Rehabilitationsmaßnahmen bei erwachsenen CI-Trägern. HNO 63(8):557–576. 10.1007/s00106-015-0037-2 26219524 10.1007/s00106-015-0037-2

